# Carbon translocation from a plant to an insect-pathogenic endophytic fungus

**DOI:** 10.1038/ncomms14245

**Published:** 2017-01-18

**Authors:** Scott W. Behie, Camila C. Moreira, Irina Sementchoukova, Larissa Barelli, Paul M. Zelisko, Michael J. Bidochka

**Affiliations:** 1 Department of Biological Sciences, Brock University, St Catharines, Ontario, Canada L2S 3A1; 2 Departamento de Entomologia, Universidade Federal de Viçosa, Viçosa, Minas Gerais 36570-900, Brazil; 3 Department of Chemistry, Brock University, St Catharines, Ontario, Canada L2S 3A1

## Abstract

*Metarhizium robertsii* is a common soil fungus that occupies a specialized ecological niche as an endophyte and an insect pathogen. Previously, we showed that the endophytic capability and insect pathogenicity of *Metarhizium* are coupled to provide an active method of insect-derived nitrogen transfer to a host plant via fungal mycelia. We speculated that in exchange for this insect-derived nitrogen, the plant would provide photosynthate to the fungus. By using ^13^CO_2_, we show the incorporation of ^13^C into photosynthate and the subsequent translocation of ^13^C into fungal-specific carbohydrates (trehalose and chitin) in the root/endophyte complex. We determined the amount of ^13^C present in root-associated fungal biomass over a 21-day period by extracting fungal carbohydrates and analysing their composition using nuclear magnetic resonance (NMR) spectroscopy. These findings are evidence that the host plant is providing photosynthate to the fungus, likely in exchange for insect-derived nitrogen in a tripartite, and symbiotic, interaction.

## Abstract (web summary)

The endophytic fungus *Metarhizium robertsii* is also an insect pathogen and can facilitate transfer of insect-derived nitrogen to host plants. Here, the authors show that carbon is transferred from plant to fungus suggesting a tripartite interaction where nitrogen is exchanged for photosynthate.

Soil fungi form intimate symbioses with over 90% of all vascular plant species^1^. These interactions have evolved over 400 million years, and are of tremendous ecological importance as they enhance plant nutrient acquisition^2^. Nitrogen is considered a critical limiting nutrient for plant growth, and generally must be fixed by nitrogen fixing bacteria for plant consumption^3^. On the other hand, there is a scarcity of easily utilizable carbon in the soil for many microbes, and subsequently, there is tremendous competition amongst them for this resource^4^. Plants often produce excess photosynthate, particularly when provided with adequate nitrogen, some of which is released into the rhizosphere^5^. These nutritional circumstances present the opportunity for a potential symbiotic exchange of nutrients between plants and endophytic, insect-pathogenic fungi; plants require nitrogen, and these fungi require accessible carbon.

There are several documented examples of nitrogen-carbon exchange in fungal-plant symbioses. Many fungal-plant symbionts, including mycorrhizal fungi, are obligate biotrophs and are therefore unable to survive outside of the plant. In these associations, the fungus acquires carbon from the plant, as it is unable to efficiently scavenge carbon from soil^6^. In exchange for plant-derived sugars, the fungus provides the host plant with limiting soil nutrients such as phosphate and/or nitrogen. This reciprocal exchange has been shown using gene expression or transporter-staining experiments^7^,^8^,^9^,^10^,^11^. While the movement of carbon has been shown with isotopic CO_2_, these experiments have often involved obligate biotrophs, or followed the flow of photosynthate into the rhizosphere^12^,^13^. The specific movement of atmospheric CO_2_ from a plant into a root-colonized fungus has previously not been reported.

*Metarhizium robertsii* and other *Metarhizium* species occupy a specialized ecological niche as endophytes as well as insect pathogens. Unlike other root colonizers (that is, mycorrhizal fungi), *Metarhizium spp.* are not obligate biotrophs, and are capable of living freely in soil, surviving as saprotrophs or on insect hosts. However, in their role as endophytes several *Metarhizium spp.* transfer insect-derived nitrogen to their plant hosts^14^,^15^. That is, *Metarhizium* is able to infect a soil-borne insect and transfer insect-derived nitrogen to plants, via fungal hyphae, through an endophytic association^14^,^15^. This transaction is likely widespread in nature as *Metarhizium* is a globally distributed soil fungus with a broad insect host range, infecting over 200 species of insects^16^,^17^. *Metarhizium* is also capable of forming endophytic associations, under laboratory and field conditions, with many plant species^14^,^18^. We hypothesized that in exchange for insect-derived nitrogen, transferred to host plants, *M. robertsii* (strain ARSEF 2,575) receives plant-derived photosynthate and thus plays a larger role in ecosystem nutrient cycling than previously thought.

Traditional mycological research has placed many soil fungi into a single class, as insect pathogens, endophytes or saprotrophs, for example. However, it is increasingly evident that soil-borne fungi may play multiple roles crucial to ecosystem health^19^,^20^,^21^. The ability of *Metarhizium* to transfer insect-derived nitrogen to its plant host, and receive photosynthetically fixed carbon, highlights the complex, and potentially unknown, roles of ubiquitous soil fungi.

Here we used ^13^CO_2_ to track the incorporation of atmospheric carbon into plant carbohydrates (haricot bean; *Phaseolus vulgaris*), and the subsequent translocation of ^13^C into fungal-specific carbohydrates (trehalose and *N*-acetylglucosamine) in the root/fungal complex. Through NMR analysis, we were able to identify these fungal compounds and found that *Metarhizium* receives photosynthetically fixed carbon from its plant host. We also report that the amount of carbon received by endophytic *Metarhizium* is increased when an insect is present in the system, reinforcing the reciprocal relationship between symbionts.

## Results

### ^13^C incorporation into fungal produced carbohydrates

**Figure 1 f1:**
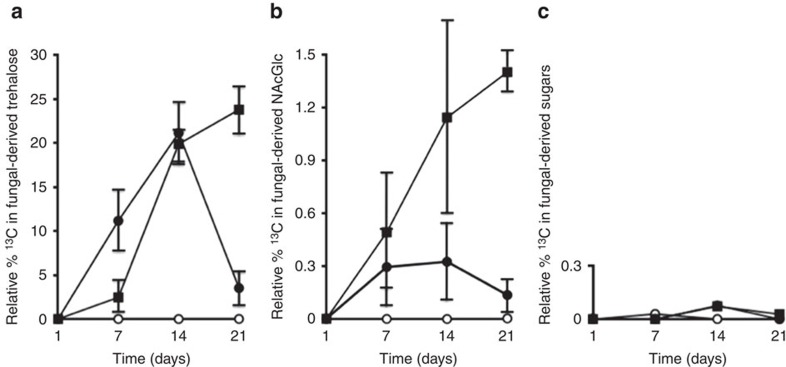
**Relative per cent** ^**13**^**C in** ***Metarhizium*** **and plant-derived sugars**. Resonances present in the ^13^C NMR spectra were integrated relative to an internal standard (100 μl MeOH, added to all samples) and then calculated as a percentage of integration value for the internal standard. (**a**) Closed circles represent ^13^C in trehalose extracted from root-colonized *Metarhizium* without an insect present. Closed squares represent ^13^C in trehalose extracted from root-colonized *Metarhizium* with insect present. Open circles represent ^13^C in trehalose extracted from roots without *Metarhizium*. (**b**) Closed circles represent ^13^C in GlcNAc extracted from root-colonized *Metarhizium* without an insect present. Closed squares represent ^13^C in GlcNAc extracted from root colonized *Metarhizium* with an insect present. Open circles represent ^13^C in GlcNAc extracted from roots without *Metarhizium*. (**c**) Closed circles represent ^13^C GlcNAc extracted from soil with *Metarhizium*, but without a plant. Closed squares represent ^13^C trehalose extracted from soil with *Metarhizium*, but without a plant. Open circles represent ^13^C in all sugars extracted from soil without *Metarhizium* and plant (*n*=20), bars represent s.e.

^13^CO_2_ was added to airtight plant growth chambers that contained plants with *Metarhizium* and insects (waxmoth larvae) in soil microcosms (Supplementary Fig. 1). The plants were separated from the insects and fungus by a 30-μm mesh. ^13^CO_2_ was provided to plants, in growth chambers, and the transfer of ^13^C into root-associated fungal biomass was assessed over a 21-day period. At 7, 14, and 21 days, in the presence of ^13^CO_2_, we observed ^13^C incorporation into *Metarhizium* (fungal-specific) carbohydrates (Fig. 1a,b). All experimental spectra demonstrated ^13^C resonances present at intensities over what was observed in natural abundance samples, indicating carbon movement from the plant to its fungal partner (Supplementary Fig. 2). Neither ^13^C labelled-trehalose nor -GlcNAc were observed in samples obtained from plants grown in the absence of *Metarhizium* (Fig. 1a,b). However, ^13^C resonances are present in the spectra representative of soluble plant sugars including fructose, glucose and sucrose.

At 21 days, in the presence of a host insect, there was a significant increase in the amount of ^13^C translocated into *Metarhizium* carbohydrates when compared with microcosms without an insect (analysis of variance, *P*<0.05) (Fig. 1a). Control plants without ^13^CO_2_ did not demonstrate ^13^C NMR resonances for carbohydrate species above natural abundance levels (Supplementary Fig. 3).

### ^13^C fixation by *Metarhizium* in soil

To ensure there was no CO_2_ fixation by *Metarhizium* in the soil, microcosms were set up with *Metarhizium* without the addition of haricot bean seedlings, and placed in assimilation chambers with ^13^CO_2_. The soil was then sampled at 1, 7, 14 and 21 days, and sequentially extracted for trehalose and GlcNAc. ^13^C-carbohydrates were not observed over what was found at natural abundance levels in soil samples (Fig. 1c) indicating that all ^13^C present in fungal biomass was obtained through the transfer of photosynthetically fixed carbon into root-colonized *Metarhizium*.

### Plant root weight

When *Metarhizium* and an insect are present, the root systems were significantly larger at both 14 and 21 days when compared to plants with *Metarhizium* only (2.9±0.8 g with versus 2.2±0.5 g without insect at 14 days (*n*=20, ±indicates s.e.), and 7.6±1.4 g with versus 5.8±0.4 g without insect at 21 days (*n*=20, ±indicates s.e.) analysis of variance, *P*<0.05). This suggests that *Metarhizium* is able to provide insect-derived nitrogen for plant-produced carbon, and thus, the root system was overall more robust.

### Root section staining

**Figure 2:**
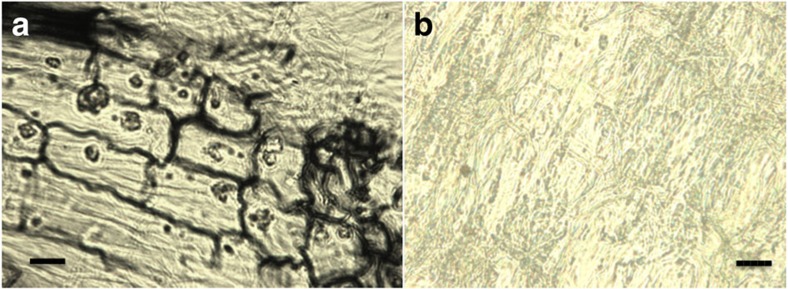
**Internal cross section of** ***M. robertsii*** **colonized haricot bean roots harvested after 10 days and stained with Chlorazol black**. (**a**) *Metarhizium* colonized plant roots, sectioned with a microtome and subsequently stained with Chlorazol black. (**b**) Plant roots grown without *Metarhizium* stained with Chlorazol black. Images were taken at × 400 magnification. scale bar, 50 μm.

Bean plant roots grown in microcosms with *Metarhizium*, under experimental conditions, were collected, sectioned, and stained with fungal-specific dye (Fig. 2). The images showed that, under current experimental conditions, *Metarhizium* was able to internally colonize plant roots.

## Discussion

**Figure 3:**
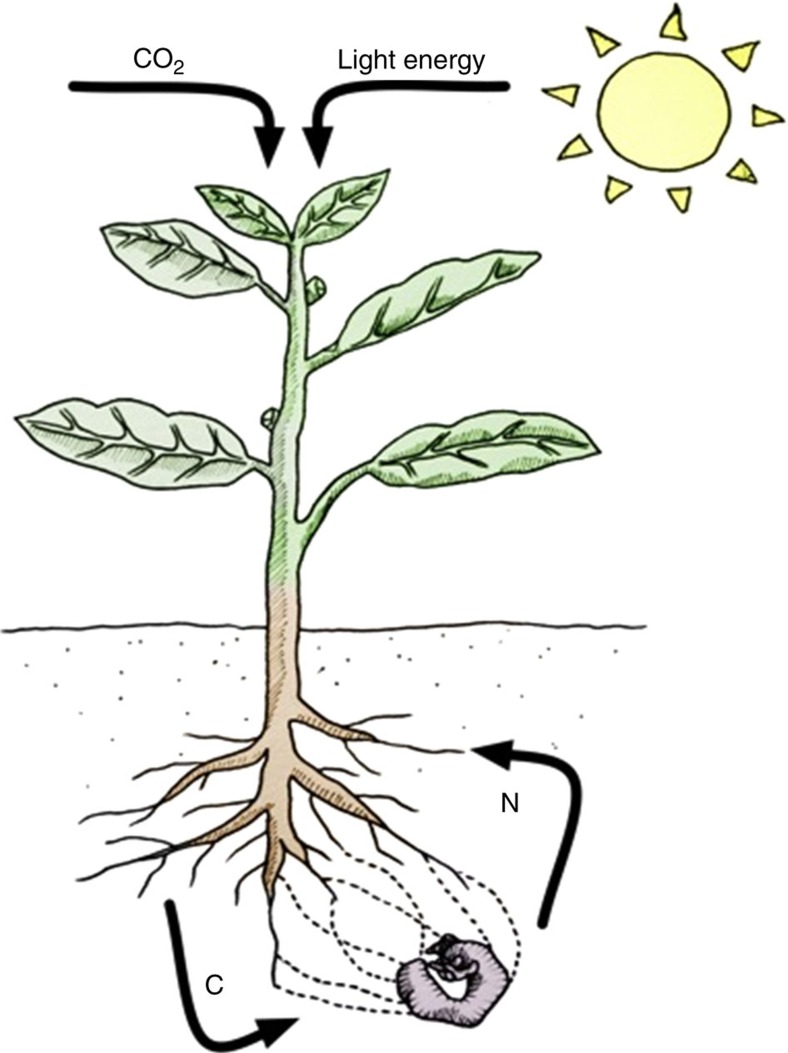
Exchange of insect-derived nitrogen for plant derived photosynthate. Representation of the exchange of insect-derived nitrogen for plant derived photosynthate between endophytic *Metarhizium* and its plant host. *Metarhizium* is able to colonize a plant root, and provide useable, insect-nitrogen in exchange for plant produced carbon.

Previously, we have shown that a host plant can derive a significant portion of its nitrogen from soil insects through an endophytic association with insect-pathogenic *Metarhizium spp*.^14^,^15^. Here we show that plants reciprocate with an exchange of photosynthate to their endophytic partners. Plants photosynthetically fix carbon, and furnish root-colonized *Metarhizium* with this carbon, while *Metarhizium* can access a source of nitrogen (that is, insects) via their specialization as insect pathogens (Fig. 3).

Plants provided carbon to endophytic *Metarhizium* even in the absence of a host insect in the microcosm (Fig. 1). However, in the presence of a host insect there was significantly greater ^13^C transfer to *Metarhizium*. A similar relationship was observed in *Medicago truncatula* roots colonized with arbuscular mycorrhizal fungi. In this symbiosis a reciprocal exchange of nutrients stabilized the root/fungal complex and the plant host increased carbon flow to *Glomus* spp. upon receiving increased nutrients from the fungal partner^22^. Therefore, the reciprocity seen in the *Metarhizium*/plant relationship may increase the overall stability of the partnership, ensuring that each symbiont is provided with adequate nutrients.

The ability of *Metarhizium* to receive plant-derived carbon, suggests the involvement of transporters that allow for fungal assimilation of photosynthate. In *Metarhizium*, an oligosaccharide transporter, *Metarhizium* raffinose transporter (*mrt*), plays a role in the uptake of raffinose from the rhizosphere^23^. However, in obligate plant/fungal relationships, there is evidence that plant synthesized monosaccharides are shuttled into the fungus directly from the root^24^. In mycorrhizal fungi, for example, there is a specific increase in the monosaccharide transporter MST2 during plant root colonization^25^. *Metarhizium* has three identified monosaccharide transporters (accession numbers: MAA_02403, MAA_03088, MAA_07773), and while these transporters have not been characterized in terms of specific function or location, BLAST analyses show sequence identities with sugar transporters in other root colonizing fungi such as *Beauveria bassiana*, and *Glomerella graminicola*, suggesting their role in root colonization and nutrient exchange.

There were no overall differences in root colonization (as measured by CFU per gram root) when *Metarhizium* and insect were present in the system, compared with only *Metarhizium* present (Supplementary Fig. 4). Bean plants have previously shown increased root biomass when *Metarhizium* was present with an insect as compared with when *Metarhizium,* or an insect, are present singly^15^. An analogous situation has been observed in arbuscular mycorrhizal fungi (AMF) where AMF-colonized roots typically lead to healthier plant systems, and an overall increase in primary production^22^. Plants that receive more insect-derived nitrogen through an endophytic association with *Metarhizium* are potentially able to allocate more resources to root growth, production and photosynthesis, the resultant photosynthate could then be used to reinforce and stabilize nutrient exchange with *Metarhizium*.

*Metarhizium* is an insect pathogenic, root colonizing fungus. These two lifestyles are linked through reciprocal nutrient exchange with a host plant, whereby the fungus receives carbon in exchange for insect-derived nitrogen. Interestingly, *Metarhizium* is phylogenetically related to other endophytes^26^ and we suggest that their evolution as insect pathogens, as well as their ability to translocate nitrogen^27^, allowed them to effectively barter a specialized nitrogen source (that is, insects) with host plants for photosynthate. While there has been previous work that has indicated the role that root colonizing fungi play in plant nutrient acquisition, the present study provides information on multi-role lifestyles of insect-pathogenic fungi in soil environments. These unique endophytes may be widespread in nature, and contribute to the overall global cycling of nutrients in a way more profound than previously known.

## Methods

### Fungal cultures and plant material

*Metarhizium robertsii* strain ARSEF 2575 was obtained from the US Department of Agricultural Research Service Collection of Entomopathogenic Fungal Cultures, Ithaca, New York. Stock cultures were grown at 27 °C on potato dextrose agar (PDA; Difco laboratories, Mississauga, Ontario, Canada. *Phaseolus vulgaris* (Haricot Bean) seeds were obtained from OSC seeds, Waterloo, Ontario, Canada. *M. robertsii* was transformed for expression of the green fluorescent protein (GFP) gene, *egfp*, utilizing the vector pFBENGFP in *Agrobacterium tumefaciens*-mediated transformation^28^.

### Seed sterilization and plating

Seeds were surface sterilized before use in order to prevent any unwanted fungal or bacterial growth. Seeds were immersed in sterile distilled water for thirty minutes in a 50 ml capped plastic tube. They were then washed in a 4% sodium hypochlorite solution three times, ten minutes each wash. The seeds were rinsed with sterile distilled water between each wash. The seeds were then placed in 15% hydrogen peroxide for thirty minutes followed by three washes with sterile distilled water. The seeds were then kept overnight at 4 °C to allow for synchronization of growth, then plated on water agar and kept at 25 °C for a photoperiod of 16 h a day for a minimum of seven days in order to obtain seedlings which were used in the study.

### Plant growth

Plants were grown in plastic garden pots (height 20 cm). The pots were sterilized with UV light for 2.5 h before use. Soil was sterilized prior to use by autoclaving 3 × on a biohazard cycle and allowed to cool between each cycle (19 h). Pots were partially filled with sterile soil, and five 1cm × 1cm agar plugs were added. Pots were then filled with soil 3 cm from the top and a seedling planted. Where appropriate, *Metarhizium* infected insects were added to soil microcosms. *Galleria mellonella* were infected with conidia by placing the larva on a 10-day-old conidiating culture of *M. robertsii*. The plate was agitated for two minutes allowing the fungus to sufficiently adhere to the insects. Insects were then placed into Petri dishes that had a circle cut into the lid ∼7 cm in diameter, which was subsequently covered with 30 μm mesh. The mesh was adhered to the Petri dish lid with silicon glue. This allowed for the movement of fungus out of the Petri dish while ensuring the insects could not interact with the plant roots. Plants were watered daily with sterile, distilled water and once a week with 50 ml of 50% MMN solution ((0.05 g CaCl_2_, 0.025 g NaCl, 0.05 g KH_2_PO_4_, 0.5 g (NH_4_)_2_PO_4_, 0.15 g MgSO_4_·7H_2_0, 1 mg FeCl_3_·6H_2_0, 5 g glucose monohydrate, 10 ml trace elements solution (3.728 g KCl, 1.546 g H_3_BO_3_, 0.845 g MnSO_4_·H_2_0, 0.05 g ZnS0_4_·7H_2_0, 0.0125, g CuS0_4_, 0.05 g (NH_4_)_6_Mo_7_0_24_·4H_2_0 per 1 l) per 1 l).

### Assimilation chamber and ^13^CO_2_ addition

Plant assimilation chambers were constructed to provide plants with a 1,500 p.p.m. ^13^CO_2_ (99% ^13^C) environment. All chambers were 60 l by volume (Supplementary Fig. 1) and were airtight. Plants were placed in the assimilation chambers and sealed, once inside the chambers, ^13^CO_2_ was added until 1,500 p.p.m. was reached. Plants were allowed to grow in a ^13^CO_2_-rich environment for 8 h, during maximum photosynthetic period. Plants were provided with ^13^CO_2_ every 48 h.

### Extraction of soluble sugars and *N*-acetylglucosamine

Soluble sugars and *N*-acetylglucosamine extractions were carried out by methanol extraction^29^. Twenty plants were collected at appropriate time points (1, 7, 14, 21 days), microcosms were dismantled, and insects analysed to ensure infection by *Metarhizium*. In these microcosms 100% of insects were observed to be infected and killed by *Metarhizium*. Dead insects were mummified with a layer of fungal hyphae and conidia. Roots were gently washed free of soil, lightly dried, and detached from the stalk with a scalpel blade and weighed. Plant roots were pooled, 5 plants per pool and lyophilized. Plant root tissue pools were frozen in liquid nitrogen and ground with mortar and pestle to a powder in 4–6 ml of MeOH/H_2_0 (70/30). The mixture was then filtered through Fischer brand P4 filter paper, and methanol was removed by evaporation under vacuum. The aqueous solution was freeze-dried and stored at 4 °C for NMR analysis. The solid plant residue was then used for *N*-acetylglucosamine extraction. Plant tissue was treated for 20 min in boiling 1 M KOH. KOH was then decanted and solids were washed in deionized water and soaked in potassium phosphate buffer (pH 6.0). One unit of chitinase, 1 unit of chitosanase and 0.1 unit of glucanase were added to the samples (each enzyme was prepared as a stock solution in potassium phosphate buffer). To prevent contamination, 0.01% sodium azide was added to each sample. Samples were then incubated at 37 °C for 7 days under continuous agitation. After enzymatic digestion, samples were centrifuged and supernatants were lyophilized and stored at 4 °C prior to NMR analysis.

### Nuclear magnetic resonance spectroscopy

All NMR spectra were recorded in D_2_O on a Bruker Ultra Shield Plus AV-600 spectrometer (^1^H at 600 MHz, ^13^C at 151 MHz) with a 5 mm Bruker BBFO probe with methanol (100 μl) as an internal reference for all spectra at 49.5 p.p.m. Acquisition parameters were the same for all samples. Four spectra, each of 27,000 scans, were acquired for each sample pool. This was considered to give sufficient signal-to-noise ratio to identify incorporation of ^13^C into major metabolites of interest. NMR spectra were analysed using the Bruker Topspin v2.0 software interface. Detailed acquisition parameters can be found in Supplementary Table 1.

### Fungus colonized root staining

Harvested roots were washed in sterile distilled water until all soil removed and then stained^30^. Roots were fixed in a 1:1:1 formalin:acetic acid:alcohol solution for 24 h. The roots were then dehydrated in increasing concentrations of ethanol (30, 50 and 70%) for 30 min, then in 90% ethanol for 60 min. After dehydration, the roots were cut into 2 cm sections and cleared in methyl salicylate for 12 h. The roots were then embedded in paraffin wax and sectioned using a microtome. The wax was removed by washing the samples with xylene for 15 min. The roots were then placed in 10% (wt/vol) KOH solution and heated at 65 **°**C for 60 min. After 1 h, the KOH was decanted and replaced with Chorazol E Black staining solution (80% lactic acid, glycerol, and distilled water with 0.1% Chlorazol black E, 1:1:1:1) and heated at 65 **°**C for 60 min.

### CFU determination from soil

One gram of soil was taken from each pot, at a distance of 1.0 cm from the root and 2.0 cm below the soil surface, and suspended in 2.0 ml of 0.01% Triton-X. The samples were vortex vigorously and 0.1 ml of suspension was plated, in duplicate, on selective YPD (2 g yeast extract, 10 g peptone, 20 g dextrose, 15 g agar, 0.01 g crystal violet, 0.2 g chloramphenicol, 0.5 g cycloheximide, and 0.3 g dodine adjusted to 1 l with distilled water). Plates were incubated at 27 **°**C for 10 days. The CFUs were recorded on day 10. To confirm colonies were that of *M. robertsii* the colonies were examined, before conidiation, for green fluorescence as the strain used (2575-GFP) is a stable lab strain expressing GFP.

### CFU determination from roots

Roots were gently washed free of soil, lightly dried and detached from the stalk with a scalpel blade. The roots were sliced into 5 mm sections and placed in a 50 ml tube and weighed. A solution of 0.01% Triton-X was added to the roots at a volume of 2:1 (that is, 2 ml 0.01% Triton-X to every 1 g of root). The roots were homogenized using a Tissue Tearor (Greiner Scientific) and 0.1 ml of the homogenate was plated, in duplicate, on selective YPD. Plates were incubated at 27 **°**C and the CFUs were recorded on day 10. Colonies were confirmed to be *M. robertsii* by visualization of GFP fluorescence.

### Data availability

The authors declare that all the data supporting the findings of this study, and any relevant subsequent information, is available within the manuscript and its supplementary files or is available from the authors by request.

## Additional information

**How to cite this article:** Behie, S. W. *et al*. Carbon translocation from a plant to an insect-pathogenic endophytic fungus. *Nat. Commun.* **8,** 14245 doi: 10.1038/ncomms14245 (2017).

**Publisher's note**: Springer Nature remains neutral with regard to jurisdictional claims in published maps and institutional affiliations.

## Supplementary Material

Supplementary Information Supplementary Figures and Supplementary Tables
