# Supplementary material for: Carbon translocation from a plant to an insect-pathogenic endophytic fungus
Source: Nat Commun. 2017 Jan 18;8:14245. doi: 10.1038/ncomms14245 (PMC5253661; doi:10.1038/ncomms14245)
Supplement: Supplementary Information — Supplementary Figures and Supplementary Tables [file ncomms14245-s1.pdf]

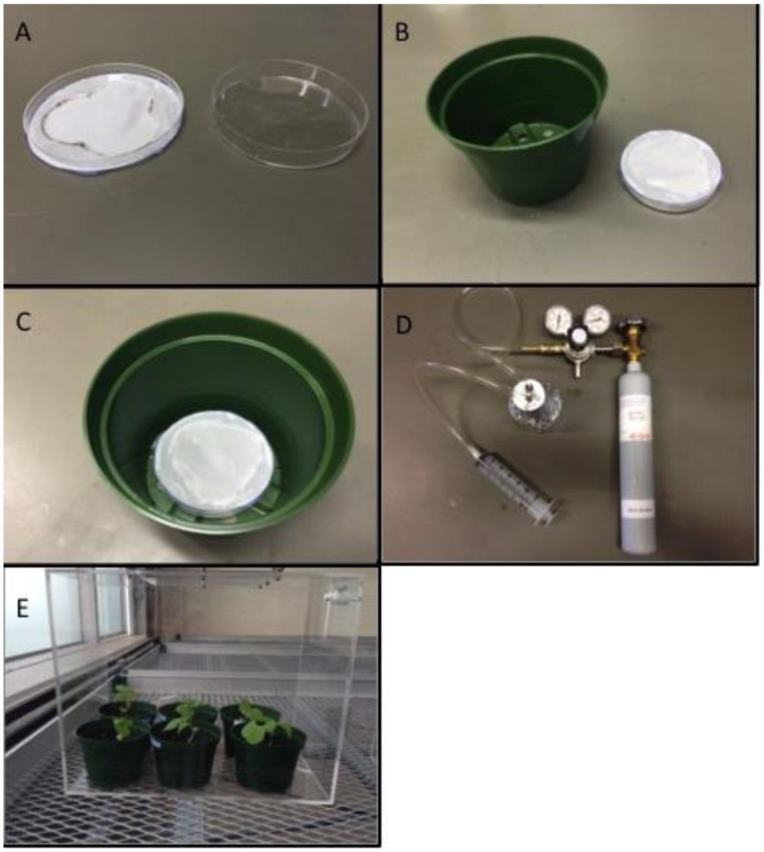

Supplementary Figure 1. Microcosm design and experimental setup. A) Empty Petri dish, with 30 micron mesh adhered to the surface with silicon adhesive. B) Closed Petri dish plant container. C) Microcosm showing Petri dish in plant container. This would be filled with soil. D) $^{13}C\ CO_2$ compressed gas bottle and gas injection syringe, used to add gas volumetrically into gas assimilation chambers. E) 7 day plants in assimilation chamber at 1500 ppm $^{13}C\ CO_2$.

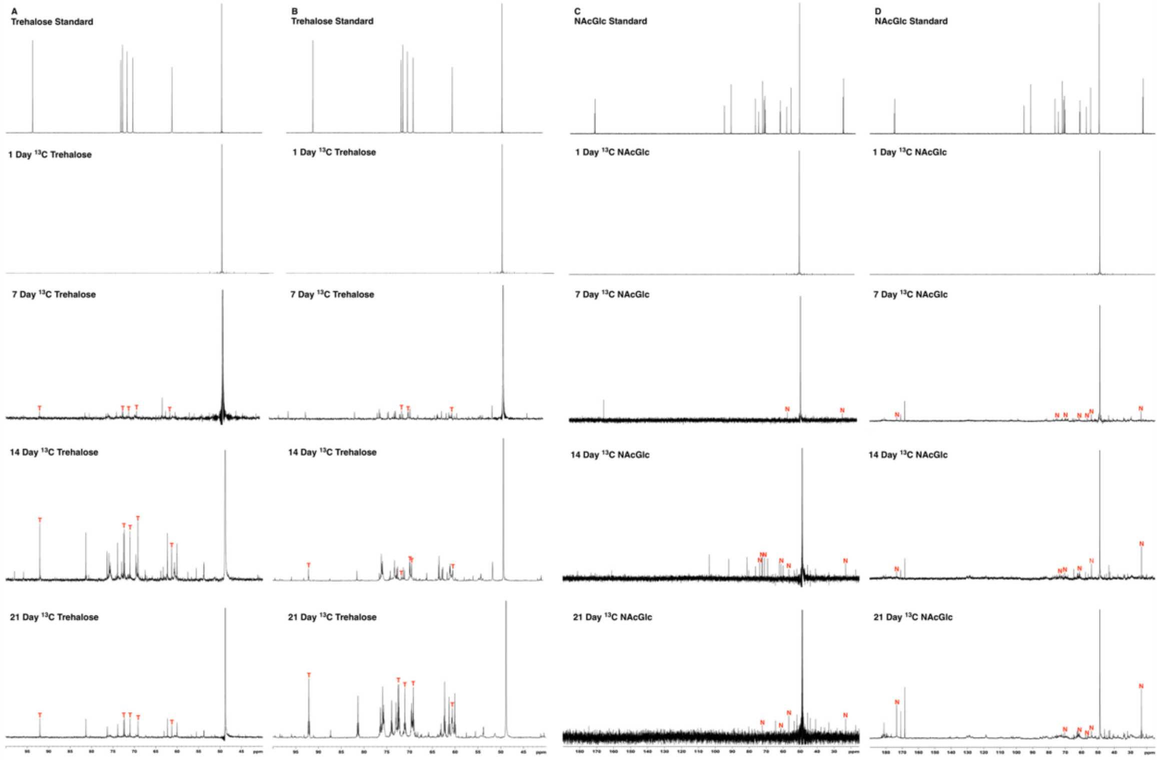

Supplementary Figure 2. ^{13}C NMR spectra of both trehalose and N-acetylglucosamine (GlcNAc) (1, 7, 14, and 21 days) obtained from *Metarhizium* colonized plant roots or *Metarhizium* colonized soil grown in ^{13}C or natural CO_2 environment, as well as control treatments. A) Trehalose spectra without insect present in soil microcosms. B) Trehalose spectra with insect present in soil microcosms. C) GlcNAc spectra without insects present in soil microcosms. D) GlcNAc spectra with insects present in soil microcosms. All spectra are comprised of 27, 000 scans, with a 100 μL MeOH internal standard (49.5 ppm).

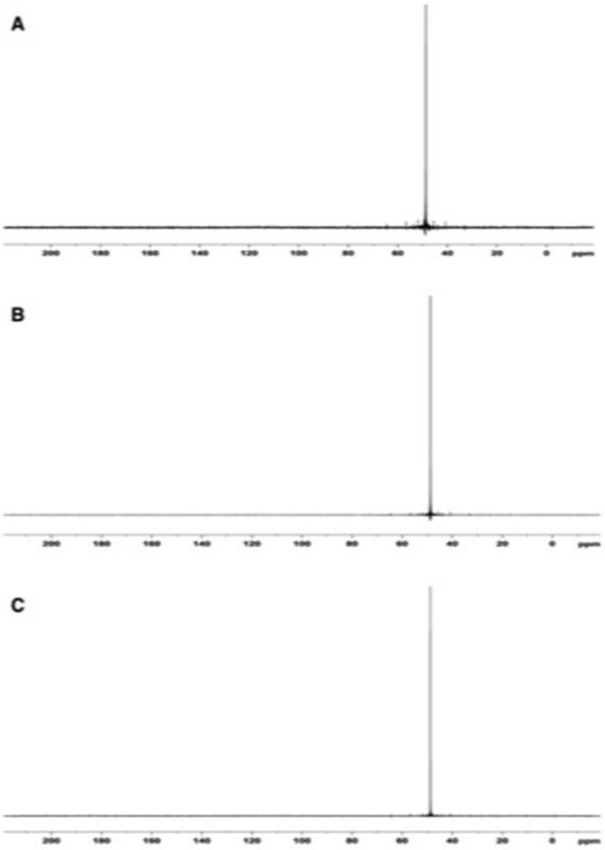

Supplementary Figure 3. ^{13}C NMR spectra of soluble sugars extracted from plants grown without ^{13}C CO_2. A) Plant grown without fungus or insect present in soil microcosms. B) Plant grown with fungus present in soil microcosms, with no insect. C) Plants grown with both fungus and insect present in soil microcosms.

A

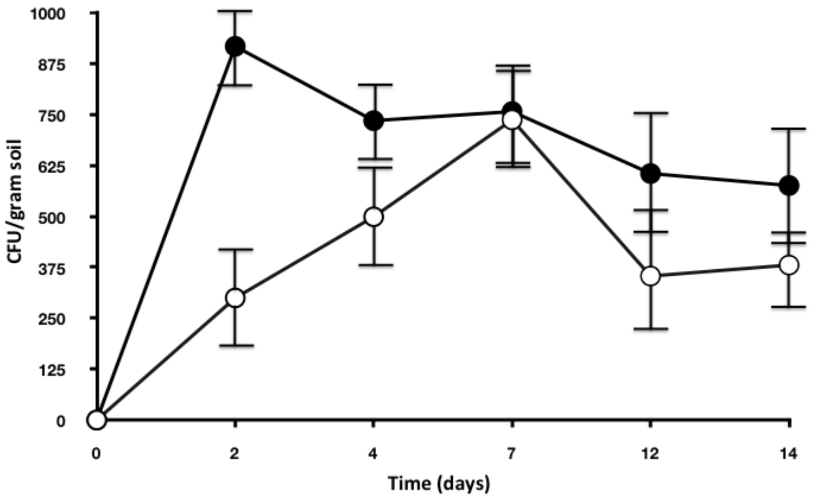

B

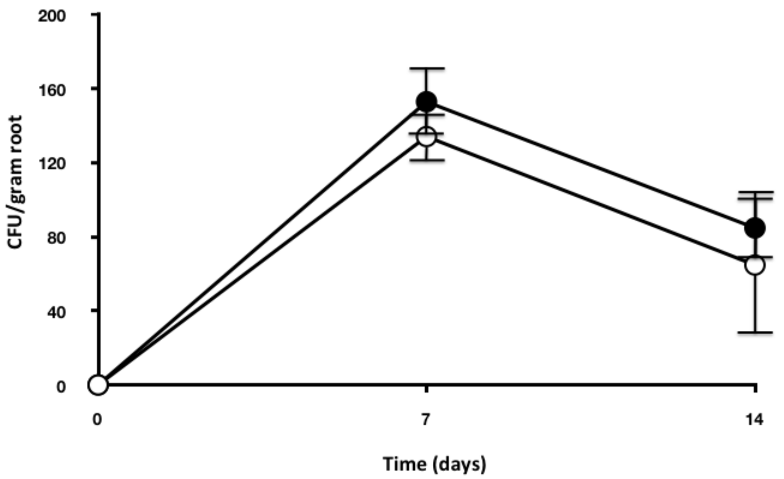

Supplementary Figure 4. A) Colony forming units/gram of soil. Closed circles represent soil samples obtained from the rhizosphere of plants grown in the presence of *Metarhizium* and an insect, open circles represent soil samples obtained from the rhizosphere of plants grown in the presence of *Metarhizium*. Soil was taken 1 cm from the plant stem at a depth of 2 cm below the soil surface. Samples were taken at 2, 4, 7, 12 and 14 days and plated on PDA. Error bars represent standard error. Significant difference (*t*-test, $p<0.05$) at day 2. B) Colony forming units/gram wet root weight. Open circles represent plants grown in the presence of *Metarhizium*, closed circles represent plants grown in the presence of *Metarhizium* and an insect. Roots were harvested at 0, 7, and 14 days and homogenized. Homogenate was plated onto PDA. No significant differences were observed.

Supplementary Table 1. Data acquisition parameters for nuclear magnetic resonance (NMR) spectroscopy.

Probe: 5mm PABBO BB
Size of FID (TD): 32768
Solvent: D2O
Number of scans (NS): 27000
Dummy scans (DS): 0
Spectral width (SWH): 35971.223 Hz
FID resolution (FIDRES): 1.09775 Hz
Acquisition time (AQ): 0.4554752 sec
Receiver gain (RG): 20642.5
Dwell time (DW): 13.900 microseconds
Pre-scan delay (DE): 6.00 microseconds
Requested probe temperature (TE): 295.1 K
Delay (D1): 2.00000000 sec
D11: 0.03000000 sec
TD0: 27

Nuc1: 13C
P1: 10.50 sec
PL1: -3.00 dB
PL1W: 146.34667969 W
SFO1: 150.9355021 MHz

Nuc2: 1H
PCPD2: 70.00 microseconds
PL2: -4.00 dB
PL12: 11.69 dB
PL13: 15.00 dB
PL2W: 31.54786682 W
PL12W: 0.85107934 W
PL13W: 0.39716411 W
SFO2: 600.2024008 MHz
